# New Species of the Fern Genus *Lindsaea* (Lindsaeaceae) from New Guinea with Notes on the Phylogeny of *L*. sect. *Synaphlebium*

**DOI:** 10.1371/journal.pone.0163686

**Published:** 2016-10-19

**Authors:** Shi-Yong Dong, Zheng-Yu Zuo, Yi-Shan Chao, Kipiro Damas, Bernard Sule

**Affiliations:** 1 Key Laboratory of Plant Resources Conservation and Sustainable Utilization, South China Botanical Garden, Chinese Academy of Sciences, Guangzhou 510650, China; 2 University of Chinese Academy of Sciences, Beijing 100093, China; 3 Department of Biomedical Science and Environmental Biology, Kaohsiung Medical University, Kaohsiung, Taiwan; 4 PNG Forest Research Institute, Lae 411, Papua New Guinea; Institute of Botany, CHINA

## Abstract

To determine the taxonomic identities and the systematic positions of some collections of *Lindsaea* sect. *Synaphlebium* (Lindsaeaceae) from Papua New Guinea, we conducted morphological comparisons and phylogenetic analyses on the whole section. A total of 22 morphological characters were selected and coded for each of all known taxa in *L*. sect. *Synaphlebium*, and were analyzed using maximum parsimony. The datasets containing either of or combined two plastid DNA sequences (*trnL-trnF* spacer and *trnH-psbA* spacer) of 37 taxa were analyzed using maximum parsimony, maximum likelihood, and Bayesian inference. Morphological comparisons revealed two new species which are formally published here as *L*. *subobscura* and *L*. *novoguineensis*. *Lindsaea subobscura* is similar to sympatric *L*. *obscura* and *L*. *modesta* but differs in the obviously reduced upper pinnules and other characters. *Lindsaea novoguineensis* is most similar to *L*. *pacifica* from Melanesia but differs in having rhomboid pinnules with truncate apices and concave soral receptacles. Molecular analyses resolved *L*. sect. *Synaphlebium* and allied species into five well-supported clades, namely *L*. *rigida* clade, *L*. *obtusa* clade, *L*. *pulchella* clade, *L*. *multisora* clade, and *L*. *cultrata* clade. The new species *L*. *novoguineensis* is included in *L*. *obtusa* clade; *L*. *subobscura* is in *L*. *pulchella* clade; whereas the majority of *L*. sect. *Synaphlebium* is clustered in *L*. *cultrata* clade. As the section *Synaphlebium* sensu Kramer is strongly suggested as polyphyletic, we propose the concept of a monophyletic *L*. sect. *Synaphlebium* in a broad sense that comprises five lineages. The morphological circumscription of *L*. sect. *Synaphlebium sensu lato* and the divergence in morphology, habit, and distribution between the five lineages are briefly discussed. Further molecular study is needed to test the systematic positions of 16 other species which are supposed to be within *L*. sect. *Synaphlebium sensu lato* but have not been included in this and previous molecular analyses.

## Introduction

*Lindsaea* Dryand. ex Sm. is a pantropical genus, with ca. 150 species mostly in seasonally moist or everwet, often submontane forests [1]. It is taxonomically well studied since Kramer [2–10] had provided a series of revisions for regions covering the whole distribution range of this genus. The diagnostic characteristics for *Lindsaea* mainly include 1) stems bearing some (never copious) narrow, basally attached scales, 2) fronds mostly with dimidiate pinnules, 3) veins free, or in some cases, anastomosing and without free veinlets included in areoles, 4) sori marginal or submarginal, and 5) indusia usually linear, parallel to and opening towards the margin. Two subgenera, *i*.*e*., terrestrial *Lindsaea* and epiphytic *Odontoloma* (Hook.) K.U. Kramer, and 21 sections under the genus had been recognized by Kramer [2–10]. *Lindsaea* and its closely allied genera, traditionally including *Odontosoria* Fée, *Ormoloma* Maxon, *Sphenomeris* Maxon, *Tapeinidium* (C. Presl) C. Chr., and *Xyropteris* K.U. Kramer, are treated as a tribe or a subfamily under Dennstaedtiaceae [1,11] or accepted as a separate family, namely Lindsaeaceae [12,13]. Molecular evidence, however, suggests the affinity of *Lindsaea* group not to dennstaedtioids but to *Saccoloma* Kaulf. (Saccolomaceae), and supports the treatment of lindsaeoids as a separate family which, together with Saccolomaceae, forms a sister clade to the remaining Polypods [14,15,16,17]. Lehtonen et al. [17] reconstructed the phylogeny of Lindsaeaceae based on a rather wide sampling and five regions of plastid DNA. The traditional *Lindsaea*, by excluding ca. five species [now placed in *Osmolindsaea* (K.U. Kramer) Lehtonen & Christenh. or *Nesolindsaea* Lehtonen & Christenh.], was strongly suggested to be a natural group and was inferred to include 13 lineages. The two subgenera (*Lindsaea* and *Odontoloma*) and most sections proposed by Kramer within *Lindsaea* were not supported to be monophyletic [17].

New Guinea, as a joint between the Malesian flora, Melanesian flora, and Australian flora, is well known for its immense biodiversity. During a collecting trip to Wagau, central Morobe Province, Papua New Guinea in December 2013, we collected some interesting ferns. Among them were taxa in the genus *Lindsaea*, represented by the collection *Dong 4016* and the *Dong 4046*, but their identities remain uncertain. Both collections can be readily recognized as members of *L*. sect. *Synaphlebium* [5,7] because they are terrestrial plants, having dimidiate pinnules and anastomosing veins. According to Kramer [6,7,8,9,18] and Lehtonen et al. [19] there are a total of 23 species currently known in *L*. sect. *Synaphlebium*, of which 16 occur in Malesia (including New Guinea) [7]. *Dong 4016* is morphologically similar to *L*. *obscura* Brause and *L*. *modesta* K.U. Kramer, two endemic species in New Guinea, but apparently different in the shape of pinnules and other characters. *Dong 4046*, on the other hand, is morphologically not similar to any known species in Malesia but somewhat similar to *L*. *pacifica* K.U. Kramer from Melanesia. To determine the true identities of the two collections, we conducted detailed comparisons of morphology as well as phylogenetic analyses of plastid DNA sequences between the two collections and all known species with dimidiate pinnules and anastomosing veins.

## Materials and Methods

### Morphological study

The local field trip was supported by PNG Forest Research Institute. The two new species in question were found in the field of Wagau, a private land in Morobe Province, Papua New Guinea. Access permission from the land owner was obtained prior to entering the private land. A total of 1169 herbarium specimens of *Lindsaea* at IBSC, LAE, and PE representing 48 species, as well as the type specimens of all species belonging to *L*. sect. *Synaphlebium* in various herbaria (mainly in B, BM, BO, K, L, MICH, and P) were carefully studied. In addition to the 23 species known in *L*. sect. *Synaphlebium*, three other species, namely, *L*. *pulchella* var. *lomatosora* Kramer, *L*. *rigida* J. Sm., and *L*. *werneri* Rosenst., also with anastomosing veins and dimidiate pinnules but were grouped into the epiphytic *L*. subgen. *Odontoloma* by Kramer [7] were included in the morphological study. To ensure that our understanding of species are correct, we checked the nomenclatural type of all species involved in the present study. A total of 22 morphological characters, most of which have been widely used to identify sections within *Lindsaea* or distinguish species within sections by Kramer [5,7], were selected to compare new collections and all the known 26 taxa with anastomosing veins and dimidiate pinnules. The states of the 22 characters were determined and numerically coded generally based on herbarium specimens, with reference to the description and/or illustrations provided by various authors [6,7,19,20]. Descriptive terms, as ‘pinnule’ exclusively refers to the ultimate free division of lamina regardless of the branching order on lamina, are in accordance with the terminology used by Kramer [2,5,7]. The distribution information of species is from the literature [2–10]. Three new collections, *L*. *obtusa* J. Sm. ex Hook. (*Dong 3936*), *Dong 4016*, and *Dong 4046*, are kept in the herbarium of South China Botanical Garden, Chinese Academy of Sciences (IBSC), with duplicates deposited in the herbarium of Forest Research Institute, Lae, Papua New Guinea (LAE). The 22 characters and their states are listed below. The morphological matrix including 22 characters and 28 taxa is presented in S1 Appendix.

1. Plants habit—(0) terrestrial, (1) epiphytic.
2. Rhizome surface—(0) covered with copious scales, (1) nearly naked.
3. Rhizome diameter—(0) less than 1 mm, (2) usually 2 mm or more.
4. Frond arrangement—(0) nearly clustered, less than 2 mm apart, (1) close, 3–5 mm apart, (2) remote, over 10 mm apart.
5. Stipe length—(0) more than 3/5 of the lamina, (1) less than 1/3 of the lamina.
6. Stipe color—(0) stramineous, (1) dark brown or blackish.
7. Rachis abaxially—(0) bi-angular, (1) terete.
8. Lamina dissection—(0) exclusively 1-pinnate, (1) exclusively 2-pinnate, (2) 1- or 2-pinnate.
9. Pinnae number—(0) less than 4, (1) 4–8 to one side of rachis.
10. Upper pinnae—(0) gradually reduced, (1) suddenly reduced, (2) not reduced at all towards apex of lamina.
11. Basal pinnules—(0) not or hardly reduced, (1) distinctly reduced.
12. Lower pinnules—(0) more widely placed, (1) not more widely placed than middle pinnules.
13. Pinnule shape—(0) triangular or flabellate, (1) rhomboid.
14. Pinnule apically—(0) truncate, (1) rounded or nearly so.
15. Pinnule length—(0) less than 2 times, (1) 2 times or more as long as wide.
16. Pinnule width—(0) narrowed, (1) hardly narrowed toward apex.
17. Incisions on pinnules—(0) shallow (not reaching the receptacles), (1) deep (over receptacles).
18. Veins pattern—(0) free or irregularly anastomosing, (1) regularly anastomosing.
19. Vein areoles—(0) none or only 1 rows, (1) 2 rows.
20. Sori—(0) continuous, (1) interrupted.
21. Sori location—(0) marginal: reaching the margin of lobes or the distance to the margin less than the width of indusia, (1) submarginal: the distance to the margin of lobes distinctly more than the width of indusia.
22. Receptacles of sori—(0) convex, (1) straight or nearly so, (2) concave.

To identify the morphologically similar species with the two unknown collections, we conducted maximum parsimony (MP) analyses of the morphological matrix using PAUP* Version 4.0d100 [21], with characters treated as unordered and equally weighted, and gaps as missing data. Trees were constructed with heuristic searches using 1,000 replicates of random stepwise addition and tree bisection and reconnection (TBR) branch swapping for parsimony criterion.

### Molecular phylogeny

#### Taxon sampling and molecular markers

The phylogenetic analyses in this study are based on the study conducted by Lehtonen et al. [17] who had reconstructed the phylogeny of the whole family Lindsaeaceae based on five regions of plastid DNA (*rpoC1*, *rps4*, *trnL-trnF* spacer, *rps4-trnS* spacer, and *trnH-psbA* spacer). This study is focused on the clade VI of *Lindsaea* in Lehtonen et al. [17] in which all sampled species with anastomosing veins and dimidiate pinnules were assembled. Hence all the 33 samples assembled in the clade VI of *Lindsaea* [17] are included in this study plus the three samples recently collected from Papua New Guinea, namely *L*. *obtusa* (*Dong 3960*), *Dong 4016*, and *Dong 4046*. *Lindsaea pectinata* was used as outgroup in our analyses as it was resolved in Clade V, being sister to Clade VI in the study of Lehtonen et al. [17]. Two plastid intergenic spacers, *trnL-trnF* and *trnH-psbA*, are herein selected to infer the phylogenetic position of the unknown taxa, since only the two markers are available for each of the 33 samples belonging to the clade VI of *Lindsaea* [17].

#### DNA extraction, amplification, and sequencing

The sequences of *trnL-trnF* intergenic spacer and *trnH-psbA* intergenic spacer for the three new collections were newly generated. For each sample the total DNA was extracted from leaf fragments preserved in silica gel using the CTAB method [22,23]. The *trnL-trnF* spacer was amplified using the “*trnL-trnF* e” and “*trnL-trnF* f” primers [24]. The primers for *trnH-psbA* spacer are “trnH^GUG^” [25] and “psbA” [26]. The PCR amplifications were performed in a total volume of 25 μL containing the following components: 1 μL total genomic DNA (10–100 ng), 2.5 μL PCR buffer (with MgCl_2_), 2 μL dNTP MIX, 0.5 μM of each primer, 0.2 μL rTaq DNA polymerase (Takara Biomedicals, Tokyo), and 18.3 μL of double distilled water (ddH_2_O). The PCR cycle for each region included an initial denaturation at 94°C for 4 min and a final extension at 72°C for 10 min. For the amplification of *trnL-trnF* or *trnH-psbA* spacer, 35 cycles were performed with the denaturation at 94°C for 1 min, annealing at 52°C for 30 sec, and the final extension at 72°C for 1 min. PCR products were sequenced with an ABI 377 automated sequencer (Applied Biosystems Inc., Foster City, CA, USA) following the manufacturer’s protocols.

The generated sequences were aligned using the CLUSTALW version 1.4 [27] and subsequently manually corrected in BioEdit 7.2.0 [28]. Ambiguously aligned regions were excluded from the analyses. Insertions and deletions (indels) were retained in the alignments. Unambiguous indels were identified and treated with “simple indel coding” method [29]. The apparently inverted fragments detected in *trnL-trnF* sequence of several samples were replaced with their complement sequences in the data sets. The sequences newly generated in this study have been deposited in GenBank, with the accession numbers listed as follows (in order of voucher, *trnL-trnF* sequence number, and *trnH-psbA* sequence number): *Dong 3690*, KU764769, KU764772; *Dong 4016*, KU764770, KU764773; and *Dong 4046*, KU764771, KU764774.

#### Data analyses

Separate and combined datasets were analyzed by maximum parsimony (MP), maximum likelihood (ML), and Bayesian inference (BI). Maximum parsimony analyses were performed using PAUP* Version 4.0d100 [21], with the same heuristic searches as running the morphological matrix. Bootstrap support (BS) for each clade was assessed using 1,000 heuristic replicates and TBR branch swapping.

For ML and BI analyses, the best-fitting models of molecular evolution were chosen based on the Akaike Information Criterion (AIC) [30] in jModeltest [31]. Analyses of ML were completed using GARLI v0.951 [32] with 2,000 bootstrap replicates and other settings at their default values. Bayesian analyses were executed in MrBayes 3.2.6 [33]. Four independent runs, each with four chains, were run for one million generations. Trees were sampled every 1,000 generations. Bayesian posterior probabilities (PP) were calculated as the majority consensus of all sampled trees after discarding the trees sampled within the burn-in phase.

### Nomenclature

The electronic version of this article in Portable Document Format (PDF) in a work with an ISSN or ISBN will represent a published work according to the International Code of Nomenclature for algae, fungi, and plants, and hence the new names contained in the electronic publication of a PLOS article are effectively published under that Code from the electronic edition alone, so there is no longer any need to provide printed copies.

In addition, new names contained in this work have been submitted to IPNI, from where they will be made available to the Global Names Index. The IPNI LSIDs can be resolved and the associated information viewed through any standard web browser by appending the LSID contained in this publication to the prefix http://ipni.org/. The online version of this work is archived and available from the following digital repositories: PubMed Central and LOCKSS.

## Results

### Morphology

**Fig 1:**
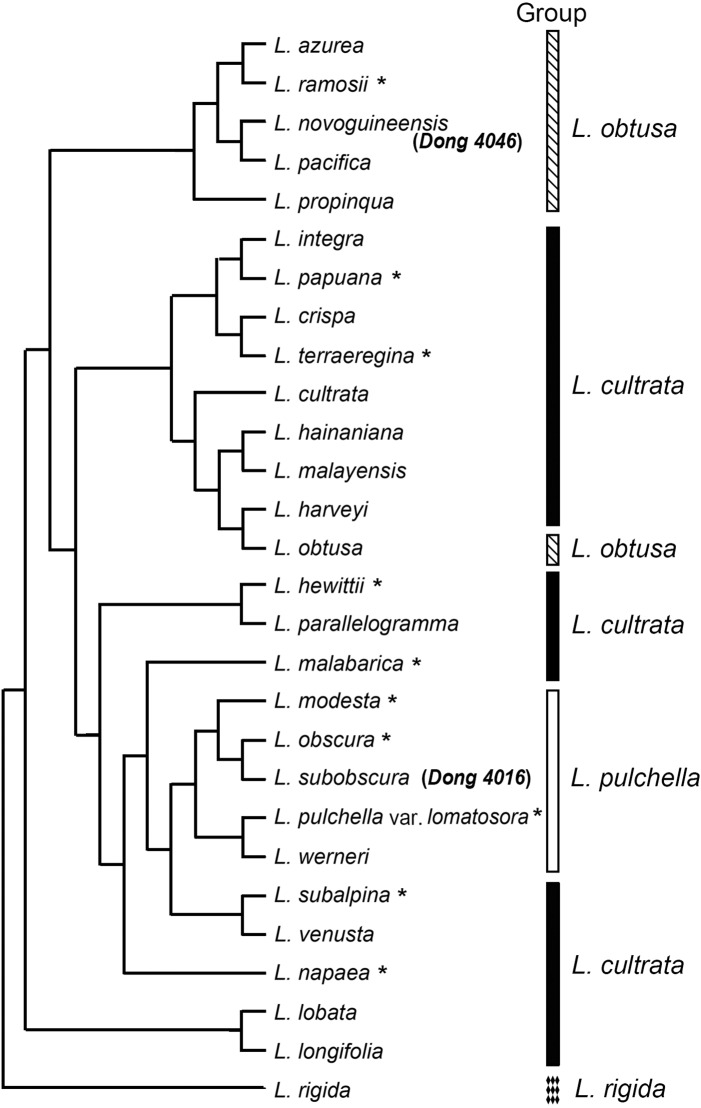
Parsimony strict consensus tree of species in *Lindsaea* with dimidiate pinnules and anastomosing veins based on 22 morphological characters. The bars in right indicate the systematic position (group) of each species inferred by molecular or morphological data. The asterisk indicates the position of the species is not confirmed by molecular data.

Of the 22 morphological characters, two are parsimony uninformative and 20 are parsimony informative. The maximum parsimony analyses of the morphological matrix produced 234 trees of 75 steps (consistency index = 0.347, retention index = 0.588, and rescaled consistency index = 0.204). One of MP trees is shown in Fig 1 where the collection *Dong 4046* is clustered with *L*. *pacifica* and the other (*Dong 4016*) is resolved sister to *L*. *obscura*. The position of *Dong 4046* suggested by MP analyses is in accordance with the direct ocular comparisons of morphology between species. *Dong 4046* is different from *L*. *pacifica* in four of the 22 characters (#4, 14, 16, and 22, see S1 Appendix). On the other hand, *Dong 4016* is different from *L*. *obscura* in three characters (#6, 10, and 13) (S1 Appendix). On the ground of the differences in several characters we conclude that the collections *Dong 4016* and *Dong 4046* each represents an undescribed species, here named *L*. *subobscura* S.Y. Dong and *L*. *novoguineensis* S.Y. Dong, respectively.

#### Description of *Lindsaea subobscura* S.Y. Dong, sp. nov.

**Lindsaea subobscura:** S.Y. Dong, sp. nov. (urn:lsid:ipin.org:names: 77157493–1)–TYPE: PAPUA NEW GUINEA. Morobe Prov.: Wagau, wet mountain forest, 1550 m, 06°51′37″S, 146°49′16″E, 18 Dec 2013, *S*.*Y*. *Dong 4016* (holotype, IBSC).

**Fig 2:**
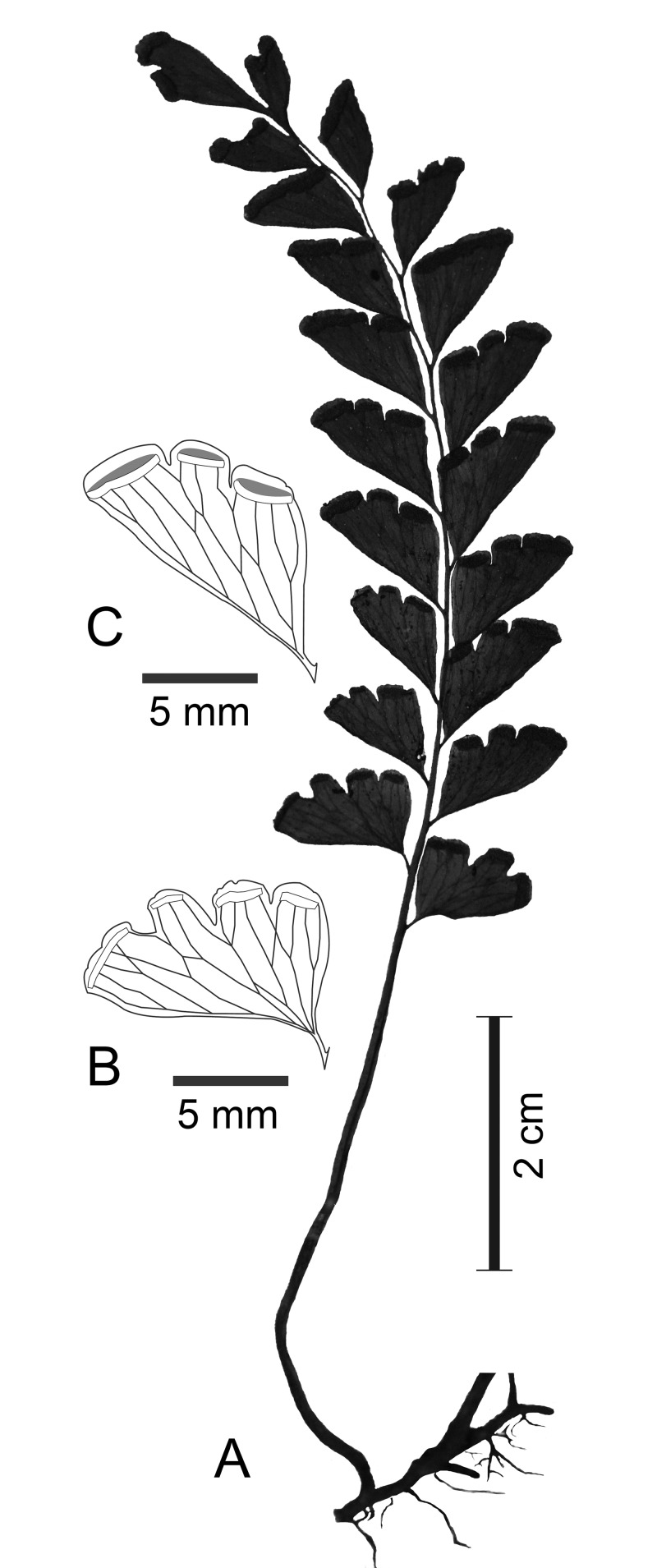
*Lindsaea subobscura* S.Y. Dong (from the holotype). A. Habit of a frond. B. A basal pinnule. C. A middle pinnule.

**Fig 3:**
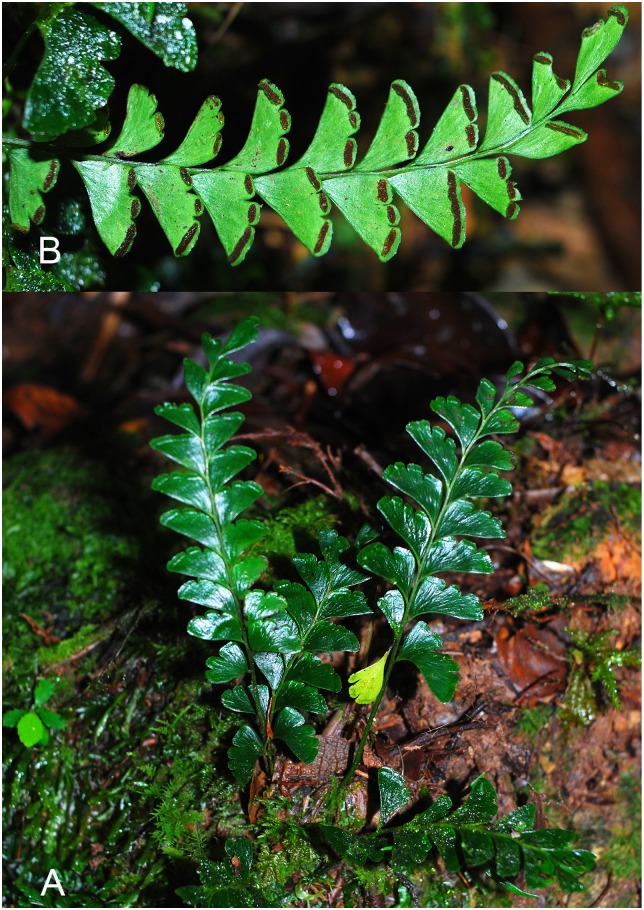
*Lindsaea subobscura* S.Y. Dong in the wild. A. Habit. B. A frond, abaxial view.

Rhizome long-creeping, wiry, ca. 1 mm in diameter, dark brown, subglabrous, covered with very few scales; scales hair-like, 2-3-seriate cells at base, ca. 1/2 mm long. Stipes 5–7 mm apart, quadrangular, sulcate adaxially, stramineous, nearly 1 mm in diameter, 2–5 cm long, obviously shorter than the lamina. Lamina (3)5–8 cm long, 2–2.8 cm wide, oblong or linear, simply pinnate, with (4)6–10 pinnules to one side of rachis; rachis similar to the stipe, stramineous, slender, quadrangular, rather deeply sulcate adaxially, bi-angular abaxially. Pinnules firmly herbaceous, dark green when dry, opaque, ascending, forming an anger of ca. 45 degree to the rachis, subtriangular, or obliquely cuneate for upper pinnules, 12–16 × 5–7 mm, acutely cuneate at base, distinctly petiolate, the petioles of pinnules 1–1.5 mm long, subacute at apex, basal pinnules the same size as or a bit larger than the next one above; lower margin of pinnules outward slightly convex, upper margin slightly to strongly convex, outer margin not developed at all, upper margin mostly with 3 rather broad and deep incisions; incisions 1–3 mm deep, the space between two lobes at margin 1–2 mm wide; upper pinnules less strongly reduced, cuneate, the terminal pinnule free, cuneate, 8–12 mm long, 3–5 mm wide at upper part, sessile, bifid at upper margin. Veins not evident, mostly once or twice forked, regularly anastomosing, forming a row of areoles along lower margin of pinnules, the outer veins free. Sori interrupted by incisions, 2–4(7) mm long, the ones on upper pinnules sometimes continuous, 4–9 mm long; receptacles straight or nearly so; indusia greenish, entire or subentire, 0.5–0.6 mm wide, rather strongly reflexed and largely overlapped by sori at maturity, not reaching the margin by the width of indusia. Figs 2 and 3.

So far only known from the type locality, terrestrial in primary evergreen forest, near a stream, rare.

*Lindsaea subobscura* is morphologically similar to *L*. *obscura* and *L*. *modesta*. The three species form a small, distinct group in *L*. sect. *Synaphlebium* characterized by the combination of simply pinnate fronds, rather slender and long-creeping rhizome, moderately arranged petioles on rhizome (5–7 mm apart), and interrupted sori. From *L*. *obscura*, the new species differs in the stramineous stipe and rachis (vs. blackish in *L*. *obscura*), subtriangular pinnules with subacute apex (vs. subtrapeziform with rounded apex in *L*. *obscura*), upper pinnules being obviously reduced (vs. hardly reduced in *L*. *obscura*), and the cuneate terminal pinnule (vs. broadly flabellate in *L*. *obscura*). *Lindsaea subobscura* differs from *L*. *modesta* mainly in the nearly naked rhizome (vs. covered with copious scales in *L*. *modesta*), upper pinnules being obviously reduced (vs. hardly reduced in *L*. *modesta*), and the pinnules much shorter and wider at base (ca. 2 times as long as wide vs. 3–4 times as long as wide in *L*. *modesta*).

#### Description of *Lindsaea novoguineensis* S.Y. Dong, sp. nov.

**Lindsaea novoguineensis:** S.Y. Dong, sp. nov. (urn:lsid:ipin.org:names: 77157494–1)–TYPE: PAPUA NEW GUINEA. Morobe Prov.: Wagau, wet mountain forest, 1200 m, 06°51′S, 146°48′E, 19 Dec 2013, *S*.*Y*. *Dong 4046* (holotype, IBSC; isotype, LAE).

**Fig 4:**
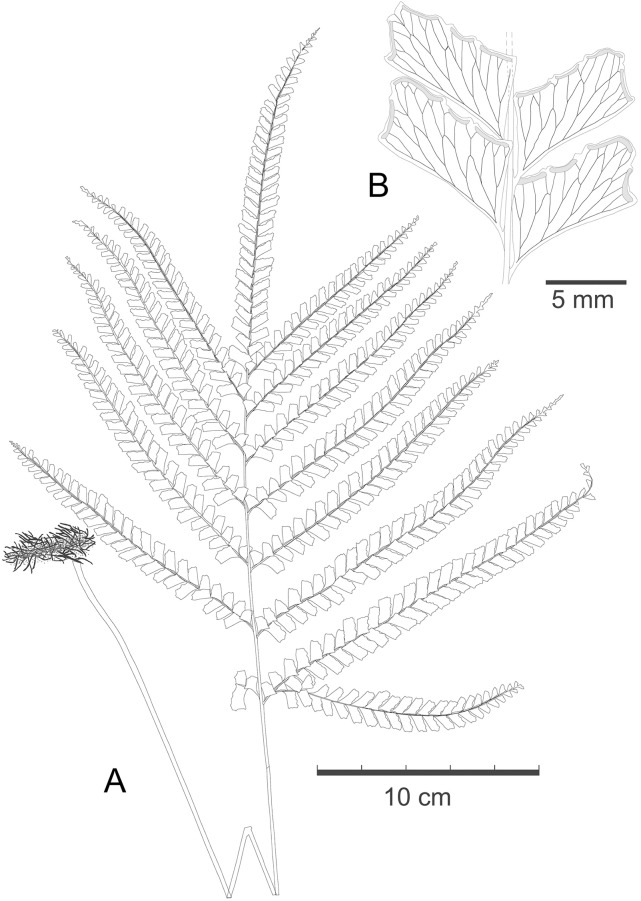
*Lindsaea novoguineensis* S.Y. Dong (from the holotype). A. Habit of a frond. B. Pinnules on middle part of a costa.

**Fig 5:**
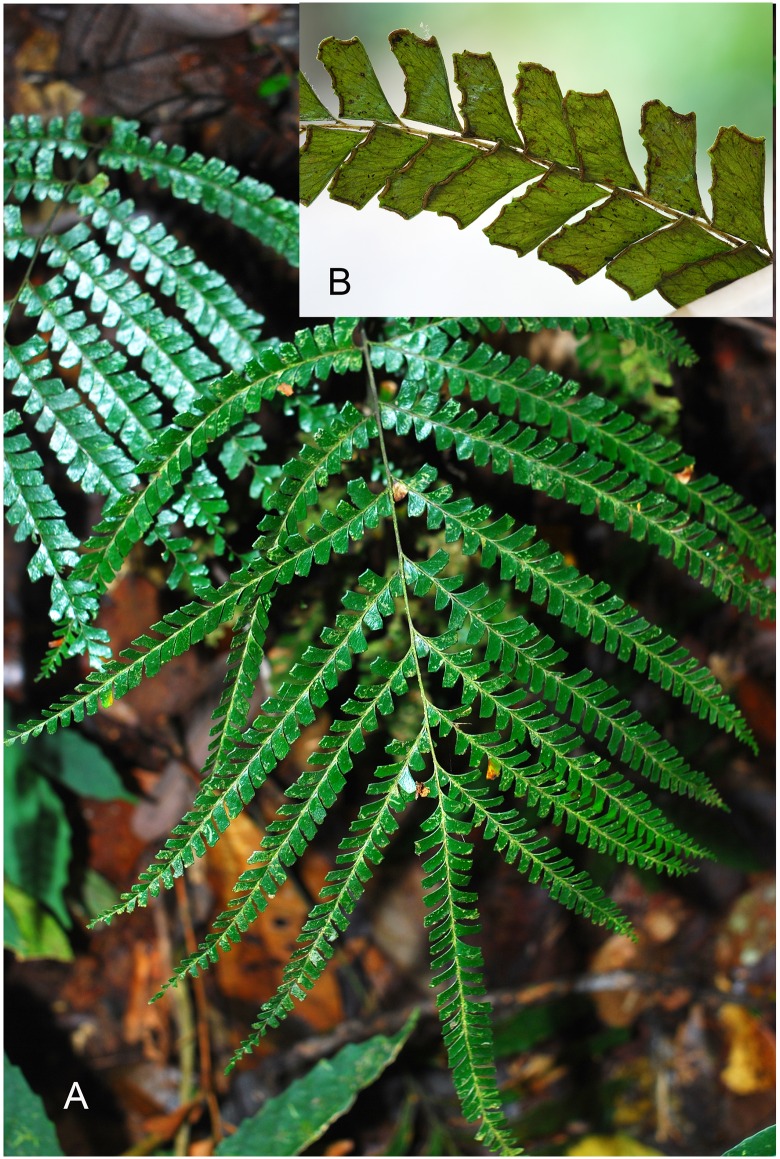
*Lindsaea novoguineensis* S.Y. Dong in the wild. A. Habit. B. Middle part of a pinna, abaxial view.

Rhizome short-creeping, wiry, ca. 3 mm in diameter, dark brown, covered with copious scales; scales linear or narrow lanceolate, dark castaneous, glossy, mostly spreading, 2–5 seriate, or sometimes to 8-seriate cells at base, 1.5–2 mm long. Stipes 5–6 mm apart, shallowly sulcate adaxially, terete abaxially, stramineous, ca. 3 mm in diameter at base, 26–33 cm long, almost as long as the lamina, with a few scales at base and glabrous above; primary rachis similar to the stipe but more deeply sulcate adaxially; secondary rachis (costa) quadrangular, rather deeply sulcate adaxially, bi-angular abaxially, stramineous. Lamina ca. 30 cm long, 20–30 cm wide, widely ovate, 2-pinnate or sometimes the basal one or two pinnae further branched on the basiscopic side near the base, with 6–7 pinnae to one side of rachis and a conform terminal one; pinnae alternate or the basal one sometimes subopposite, obliquely ascending, 12–18 cm long, 1.5–1.8 cm wide, oblong or linear, widest in the middle, slightly narrowed towards base, gradually and strongly narrowed to the long-acuminate apex. Pinnules 25–30 to a side of costa, obliquely ascending, forming an angle of ca. 60 degree to the rachis, close but hardly continuous, slightly more remotely apart on basal and apical part of costal rachis, firmly herbaceous or chartaceous, dark green when dry, transparent, mostly rhomboid, of equal width from base to apex, truncate at apex, the larger ones 8–9 mm long and 4–5 mm wide, stalks short, up to ca. 1 mm long; basal acroscopic 2–5 pinnules more or less reduced and flabellate; upper pinnules gradually and strongly reduced; the four margins of pinnules all well developed, straight or nearly so, shallowly incised on upper and outer margins, mostly with 2 incisions on upper margin and 1 on outer margin, incisions not reaching the level of soral receptacles. Veins slightly raised, evident abaxially, mostly once or twice forked, regularly anastomosing, forming a row of areoles along lower margin of pinnules. Sori interrupted, mostly 1 on outer margin and 3 on upper margin, 2–3 mm long, on 3–6(8) ends of veinlets; receptacles mostly distinctly concave; indusia greenish, entire or subentire, 0.3–0.4 mm wide, reaching or almost reaching the margin of pinnule-lobes. Figs 4 and 5.

So far only known from the type locality, terrestrial in primary evergreen forest, not common.

*Lindsaea novoguineensis* resembles *L*. *pacifica* in many characters, such as scaly and thick rhizomes, the relatively long and stramineous stipe, 2-pinnate lamina, quite a few lateral pinnae, the unreduced and equidistantly arranged lower pinnules, the regularly anastomosing veins, the rhomboid and shallowly incised pinnules, and the marginal and interrupted sori. However, *L*. *novoguineensis* differs from the latter mainly in the shape of pinnules (the same width at apex as at base, truncate at apex vs. more or less narrowed toward apex, rounded at apex in *L*. *pacifica*) and the soral receptacles distinctly concave on outer and upper margins of pinnules (vs. straight in *L*. *pacifica*). Within the scope of New Guinea, *L*. *novoguineensis* is somewhat similar to *L*. *obtusa* in the dissection of lamina, veins pattern, and the feature of sori, but apparently different from the latter by having more pinnae (6–7 pinnae vs. 1–3 lateral pinnae to one side of rachis in *L*. *obtusa*) and its unique shape of pinnules.

### Molecular study

The characteristics of the sequences for each analysis (individual *trnL-trnF* intergenic spacer, *trnH-psbA* intergenic spacer, and the two combined) are summarized in Table 1.

**Table 1:** Statistics for the datasets analyzed in this study.

	*trnL-trnF* spacer	*trnH-psbA* spacer	Combined
Accessions	37	37	37
Aligned sequence length	457	453	910
Indel characters	13	20	33
Variable characters	99	57	156
Parsimony informative characters	66	32	98
Most parsimonious trees	1,078	80	404
length of best MP trees	147	82	217
Consistence Index	0.7347	0.7195	0.7696
Retention Index	0.8375	0.8589	0.8759
BI&ML substitution model	GTR+G	GTR+G	GTR+G
Maximum likelihood score	-1,485.1653	-996.2974	-2,782.8629

**Fig 6:**
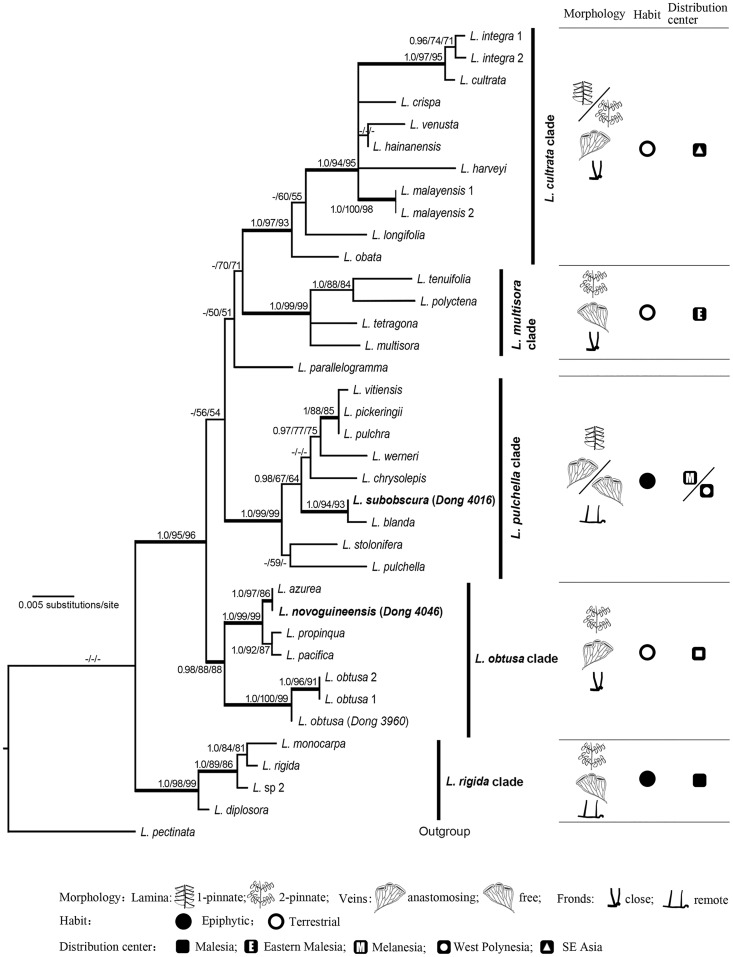
Maximum likelihood phylogram of *Lindsaea* sect. *Synaphlebium sensu lato* obtained from combined *trnL-trnF* spacer and *trnH-psbA* spacer, associated on the right with the information of morphology, habit, and distribution center for the five clades. Numbers at branches are Bayesian posterior probabilities, bootstrap values of maximum likelihood, and that of maximum parsimony, respectively. Only values over 50% for bootstrap and 0.95 for posterior probabilities are shown. Thick branches are highly supported (Bayesian PP > 0.95, MLBS > 70%, and MPBL > 70%). The two new species are in bold.

Except the position of *L*. *parallelogramma*, there is no conflict between the topologies resulting from the analyses of single *trnL-trnF*, *trnH-psbA*, or the combined dataset. The analyses based on *trnL-trnF* resolve *L*. *parallelogramma* to be sister to a big clade including most samples (just excluding *L*. *rigida* and allied three samples). Whereas in the trees based on *trnH-psbA* or combined *trnL-trnF* and *trnH-psbA*, *L*. *parallelogramma* is clustered with *L*. *cultrata* group and *L*. *multisora* group (Fig 6). The phylogenetic trees based on the combined dataset (S2 Appendix) are better resolved and better supported than those based on either *trnL-trnF* or *trnH-psbA*. The topology inferred from either ML, MP, or BI based on the combined dataset is very similar. We adopt the 50% majority-rule tree (Fig 6) inferred from ML analysis of combined *trnL-trnF* and *trnH-psbA* as a base to describe and discuss the relationships within *L*. sect. *Synaphlebium*.

The phylogeny of *L*. sect. *Synaphlebium* in broad sense (here referring to all in-group taxa) features five well-supported clades (Bayesian PP > 0.99, MLBS and MPBS > 85%): *L*. *rigida* clade, *L*. *obtusa* clade, *L*. *pulchella* clade, *L*. *multisora* clade, and *L*. *cultrata* clade (Fig 6). The first split separates *L*. *rigida* clade from other four clades, the latter four together with *L*. *parallelogramma* forming a well-supported clade (Bayesian PP = 1.0, MLBS = 98%, and MPBS = 97%). In the large, well-supported clade, the *L*. *obtusa* clade is strongly supported sister to the remaining species. Subsequently, *L*. *pulchella* clade is weakly supported sister to the clade comprising *L*. *multisora* clade, *L*. *cultrata* clade, and *L*. *parallelogramma*. The sister relationship between *L*. *multisora* clade and *L*. *cultrata* clade is similarly weakly supported.

The *L*. *rigida* clade comprises three species (*L*. *rigida*, *L*. *diplosora* Alderw., and *L*. *monocarpa* Rosenst.) and a sample with its identity unknown (*L*. sp. 2), all from Malesia (including New Guinea) [17]. The three species clustered here are all epiphytic and have 2-pinnate fronds and long-creeping rhizomes. They are not species in *L*. sect. *Synaphlebium* sensu Kramer but belong to *L*. sect. *Lindsaenium* which is different from the former by the epiphytic habit [5,7].

The next diverging clade, *L*. *obtusa* clade, consists of five species mainly from New Guinea and Pacific islands (Solomon, Fiji, east to Society Islands). *Lindsaea obtusa* represented by three samples is well resolved to be sister to the remainder (*L*. *azurea* Christ, *L*. *novoguineensis*, *L*. *pacifica*, and *L*. *propinqua* Hook.). The new species, *L*. *novoguineensis*, is strongly suggested to be sister to *L*. *azurea*; the latter is distinctly different from the former in having continuous sori. The two Malesian species form a clade sister to two Melanesian species (*L*. *pacifica* and *L*. *propinqua*). The five species in *L*. *obtusa* clade are typical members in *L*. sect. *Synaphlebium* sensu Kramer. They are all terrestrial species with 2-pinnate laminae and short-creeping rhizomes. In morphology they are very similar to some species (e.g., *L*. *harveyi* Carruth. ex Seem., Fig 1) in *L*. *cultrata* clade where the majority of *L*. sect. *Synaphlebium* is clustered. Although there is hardly any difference between the two clades in morphology, molecular data clearly suggested the clade *L*. *obtusa* represents a separate lineage from the majority of *L*. sect. *Synaphlebium* sensu Kramer (*L*. *cultrata* clade).

The next diverging lineage is *L*. *pulchella* clade, which consists of nine species mainly from the Pacific Islands (Solomon, Fiji, Samoa, etc.). The new species, *L*. *subobscura*, which is supposed to be a member in *L*. sect. *Synaphlebium* based on the terrestrial habit and anastomosing veins, is unexpectedly resolved with high support values in this clade. Except for *L*. *subobscura*, other eight species are all members of *L*. sect. *penna-arborea* featured by the epiphytic habit. *Lindsaea subobscura* is well resolved to be sister to *L*. *blanda*, an epiphytic species with free veins. Except for the different habit (terrestrial), *L*. *subobscura* is similar to other species in *L*. *pulchella* clade in the simply pinnate lamina and long-creeping rhizomes.

The *L*. *multisora* clade consists of four species mainly from Malesia (including New Guinea). This is a small natural group recognized by Kramer [2,7] as *L*. sect. *Temnolindsaea*. The synapomorphic characters for this clade include the terrestrial habit, 2-pinnate lamina, free veins, and interrupted sori. The hypothesis that *L*. sect. *Temnolindsaea* is closely related to *L*. sect. *Synaphlebium* [7] is here supported by molecular data as sect. *Temnolindsaea* and sect. *Synaphlebium* (excluding *L*. *obtusa* and allied species) are resolved as sisters. The main difference between *L*. *multisora* clade and its sister, *L*. *cultrata* clade, is the venation pattern, where veins are free in the *L*. *multisora* clade but anastomosing in the *L*. *cultrata* clade.

The *L*. *cultrata* clade is composed of nine species mainly from SE Asia (including Ceylon, S India, Indochina, S China, and Malesia). All these species are members of *L*. sect. *Synaphlebium* sensu Kramer characterized by the terrestrial habit, dimidiate pinnules, and anastomosing veins. The internal relationships of this clade are not well resolved.

The position of *L*. *parallelogramma* is different on the topology based on single *trnL-trnF* sequence when compared with the topology based on single *trnH-psbA* or the combined two sequences; in each case the topological position of the species is poorly supported. We have tried the analyses of a reduced 15-taxa dataset which includes combined five DNA regions (*rpoC1*, *rps4*, *trnL-trnF*, *rps4-trnS*, and *trnH-psbA*) for 14 accessions and combined four regions (lacking *rpoC1*) for *L*. *parallelogramma*. All these sequences are from GenBank provided by Lehtonen et al. [17]. The analyses resolve *L*. *parallelogramma* within the *L*. *cultrata* clade. *Lindsaea parallelogramma* alone forms a clade sister to the remainder species in *L*. *cultrata* clade (not shown in this paper). The position of *L*. *parallelogramma* within *L*. *cultrata* clade is also supported by the morphological similarity as *L*. *parallelogramma* is a member in traditional *L*. sect. *Synaphlebium* Kramer [7].

## Discussion

### The difference of topology between the present and a previous study

The phylogeny of *L*. sect. *Synaphlebium sensu lato* carried out in this study is mostly in accordance with the topology within the clade VI in Lehtonen et al. [17] where the authors used more markers (but with missing data) and drastically different analytical methods. The major differences involve the position of *L*. *obtusa* and that of *L*. *longifolia*. The species *L*. *obtusa* was clustered, with low support, within a clade comprising *L*. *multisora* group and *L*. *cultrata* group in the study of Lehtonen et al. [17], but is well supported sister to *L*. *pacifica* and allied species (*L*. *azurea*, *L*. *novoguineensis*, and *L*. *propinqua*) in a separate *L*. *obtusa* clade in this study (Fig 6). On the other hand, *L*. *longifolia* was resolved within the weakly supported *L*. *multisora* clade in Lehtonen et al. [17], but is strongly suggested to be a member in the well supported *L*. *cultrata* clade in this study. Our result on the position of *L*. *longifolia* in *L*. *cultrata* group is coincident with the position suggested by morphological evidence because *L*. *longifolia* is one member in *L*. sect. *Synaphlebium* characterized by the terrestrial habit, anastomosing veins, and dimidiate pinnule [7]. The close affinity of *L*. *obtusa* to *L*. *pacifica* is also readily interpreted by their morphological similarity.

### The molecular data lending support to the establishment of the new species *L*. *novoguineensis*

The analyses of the morphological characters show that the new species *L*. *novoguineensis* is most similar to *L*. *pacifica* (Fig 1), a species in Melanesia and Polynesia [6]. Compared with sympatric species in Malesia, as we have mentioned above, *L*. *novoguineensis* is most similar to *L*. *obtusa*. Our analyses of molecular data generally support the close affinity of *L*. *novoguineensis* to *L*. *pacifica* and *L*. *obtusa*, as the three species are resolved in a well-supported clade (*L*. *obtusa* clade, Fig 6). Unexpectedly, the closest species to *L*. *novoguineensis* is strongly suggested to be *L*. *azurea* by molecular data; the latter was also recorded in New Guinea [7] but distinctly differs in having continuous sori (vs. interrupted in *L*. *novoguineensis*) and 2–4 pairs of lateral pinnae (vs. 6–7 in *L*. *novoguineensis*). The evidence from molecular phylogeny and morphology supports the distinctness of *L*. *novoguineensis* among the other species of *Lindsaea*.

### The phylogenetic position of the new species *L*. *subobscura* and its systematic implications

The new species *L*. *subobscura* is supposed to be a member in *L*. sect. *Synaphlebium sensu stricto* on the ground that the species possesses the diagnostic features for the section, such as the terrestrial habit, fronds with dimidiate pinnules, and anastomosing veins [5,7]. Whereas, the molecular data strongly support the close affinity of *L*. *subobscura* to *L*. *blanda* in *L*. sect. *Penna-arborea* K.U. Kramer, which is characterized by the epiphytic habit, slim and long-creeping rhizome, simply pinnate lamina, free or anastomosing veins, and interrupted sori [5,7]. The section *Penna-arborea* was well sampled (eight of total 11 species) and was strongly supported to be monophyletic in Lehtonen et al. [17]. Our analyses also strongly support the monophyly of *L*. sect. *Penna-arborea* and reveal that *L*. *subobscura* is a member of this group (in *L*. *pulchella* clade, Fig 6). Since *L*. *subobscura* is unambiguously a terrestrial species, the character of habit shows a reversal from epiphytic to terrestrial in *L*. *subobscura* if the epiphytism is postulated to be synapomorphic for the *L*. *pulchella* clade (*L*. sect. *Penna-arborea*). Morphologically the most similar species to *L*. *subobscura* are *L*. *obscura* and *L*. *modesta* (Fig 1) which are not sampled in this study due to no materials available. Based on the close affinity to *L*. *subobscura* suggested by morphology, we suppose that the position of either *L*. *obscura* or *L*. *modesta* is in the clade *L*. *pulchella* but not in *L*. sect. *Synaphlebium s*. *s*. as so placed by Kramer [7].

### The polyphyly of *Lindsaea* sect. *Synaphlebium* in the strict sense

As pointed out by Lehtonen et al. [17], most sections of *Lindsaea* proposed by Kramer [2,3,5,8,10] were not supported monophyletic by molecular data. The section *Synaphlebium* sensu Kramer [5,7] characterized by the terrestrial habit, anastomosing veins, and dimidiate pinnules, is strongly suggested to be a polyphyletic group. Based on the terrestrial habit and other morphological similarity, *L*. *subobscura* should be placed in the section *Synaphlebium* sensu Kramer, but the molecular data strongly suggest the position of *L*. *subobscura* being in the epiphytic *L*. sect. *Penna-arborea*. In addition, *L*. *obtusa* and its allied species (*L*. *pacifica*, *L*. *propinqua*, *L*. *azurea*, and *L*. *novoguineensis*) are suggested representing a separate lineage from the majority of *L*. sect. *Synaphlebium s*. *s*. (*L*. *cultrata* clade, Fig 6), although there is morphologically no clear difference between *L*. *obtusa* group and *L*. *cultrata* group. The sister group of the clade *L*. *cultrata* is suggested to be *L*. sect. *Temnolindsaea* (*L*. *multisora* clade in Fig 6) featured by having free veins and pinnules that mostly finely and deeply dissected. This result indicates the polyphyly of *L*. sect. *Synaphlebium* in the strict sense which comprises members derived from different lineages. Neither anastomosing veins nor dimidiate pinnules is proven homologous for the strictly circumscribed *L*. sect. *Synaphlebium*. In taxonomic treatment, the *L*. sect. *Synaphlebium* in the broad sense is preferable, which should incorporate *L*. sect. *Penna-arborea* sensu Kramer [5], *L*. sect. *Temnolindsaea* sensu Kramer [2,5], and *L*. sect. *Lindsaenium* sensu Kramer [5,7]; the last one was represented by four species in phylogenetic analyses and was strongly supported sister to the remainder of *L*. sect. *Synaphlebium sensu lato* [17].

### The recognition and subdivision of *L*. sect. *Synaphlebium* in the broad sense

To morphologically recognize a species in or not in *L*. sect. *Synaphlebium s*. *l*. suggested by molecular data is really a challenge in taxonomy. As far as we know, the species now supported by molecular evidence in the monophyletic *L*. sect. *Synaphlebium* were grouped into four different sections by Kramer [2,5,7]: *L*. sect. *Synaphlebium*, *L*. sect. *Penna-arborea*, *L*. sect. *Temnolindsaea*, and *L*. sect. *Lindsaenium*. Except for the two features, i.e., dimidiate pinnules and trilete spores, other characters are all variable for the monophyletic *L*. sect. *Synaphlebium*. So it is hardly feasible to clearly define *L*. sect. *Synaphlebium s*. *l*. using a few characters. However, the characters to circumscribe the traditional four sections by Kramer could be used to recognize species of *L*. sect. *Synaphlebium s*. *l*., which are concluded here as: all species having dimidiate pinnules and trilete spores, in addition, 1) terrestrial habit and anastomosing veins; or 2) terrestrial habit, free veins, bipinnate lamina, and interrupted sori; or 3) epiphytic habit, filiform (mostly less than 1 mm thick) and deciduously scaly rhizome, and simply pinnate lamina; or 4) epiphytic habit, 1–2 mm or more thick rhizome, scales that are persistent on rhizomes, bipinnate lamina, and interrupted sori.

As molecular analyses reveal five lineages in *L*. sect. *Synaphlebium s*. *l*., it is reasonable to accept five groups within the monophyletic section, *i*.*e*., *L*. *rigida* group, *L*. *obtusa* group, *L*. *pulchra* group, *L*. *multisora* group, and *L*. *cultrata* group. It seems impossible to clearly delimitate each group and sharply distinguish them in morphology. However, we can detect divergence between the five groups in morphology, habit (terrestrial or epiphytic), and geographical distribution. For each group the main features in morphology and in ecology, the distribution center, and the known species are summarized in Table 2 and are briefly shown in Fig 6. A total of 47 species are recognized in *L*. sect. *Synaphlebium s*. *l*., of which 16 (labelled with asterisks in Table 2) have not been included in any molecular analyses due to lack of appropriate materials. The positions of the 16 unsampled species are inferred based on data from morphology, habit, and phytogeography. Further molecular studies are needed to confirm the systematic positions of the 16 species.

**Table 2:** A summary of the divergence between five groups within *Lindsaea* sect. *Synaphlebium s*. *l*. in morphology, ecology, and geographical distribution.

	Morphology	habit	Distribution center	Species
*L*. *rigida* group	Lamina 2-pinnate, veins free or irregularly anastomosing, fronds remote (2–10 cm apart)	Epiphytic	Eastern Malesia	*L*. *diplosora*, *L*. *monocarpa*, *L*. *rigida*, *L*. microstegia*, *L*. regularis*, *L*. rosenstockii*, *L*. versteegii*
*L*. *obtusa* group	Lamina 2-pinnate, veins anastomosing, fronds close	Terrestrial	Melanesia	*L*. *azurea*, *L*. *novoguineensis*, *L*. *obtusa*, *L*. *pacifica*, *L*. *propinqua*, *L*. ramosii*
*L*. *pulchella* group	Lamina 1-pinnate, veins free or anastomosing, fronds remote (1–5 cm apart)	Mostly Epiphytic	Melanesia and West Polynesia	*L*. *blanda*, *L*. *chrysolepis*, *L*. *pickeringii*, *L*. *pulchella*, *L*. *pulchra*, *L*. *stolonifera*, *L*. *subobscura*, *L*. *vitiensis*, *L*. *werneri*, *L*. modesta*, *L*. obscura*, *L*. roemeriana*
*L*. *multisora* group	Lamina 2-pinnate, veins free, fronds close	Terrestrial	Malesia	*L*. *multisora*, *L*. *polyctena*, *L*. *tenuifolia*, *L*. *tetragona*, *L*. kingii*, *L*. natunae*
*L*. *cultrata* group	Lamina 1- or 2-pinnate, veins anastomosing, fronds close	Terrestrial	SE Asia	*L*. *crispa*, *L*. *cultrata*, *L*. *integra*, *L*. *harveyi*, *L*. *hainaniana*, *L*. *lobata*, *L*. *longifolia*, *L*. *malayensis*, *L*. *parallelograma*, *L*. *venusta*, *L*. hewittii*, *L*. malabarica*, *L*. napaea*, *L*. papuana*, *L*. subalpina*, *L*. terraeregina*

* indicates the species which have not been subjected to molecular analyses.

## Supporting Information

S1 Appendix Morphological data matrix of *Lindsaea* species with anastomosing veins and dimidiate pinnules. (DOCX)

S2 Appendix Alignment matrix of combined *trnL-trnF* intergenic spacer and *trnH-psbA* intergenic spacer of 37 samples used to infer the phylogeny presented in Fig 6. (TXT)

## References

[1] Kramer KU, Green PS. Pteridophytes and Gymnosperms In: Kubitzki K, editors. The families and genera of vascular plants. Berlin: Springer-Verlag; 1990 pp. 1–404.

[2] Kramer KU. A revision of the genus *Lindsaea* in the New World with notes on allied genera. Acta Bot Neerl. 1957; 6: 97–290. 10.1111/j.1438-8677.1957.tb00576.x

[3] Kramer KU. The lindsaeoid ferns of the Old World I. New Caledonia. Acta Bot Neerl. 1967; 15: 562–584. 10.1111/j.1438-8677.1966.tb00256.x

[4] Kramer KU. The lindsaeoid ferns of the Old World II. A revision of *Tapeinidium*. Blumea. 1967; 15: 545–556.

[5] Kramer KU. The lindsaeoid ferns of the Old Word III. Notes on *Lindsaea* and *Sphenomeris* in the Flora Malesiana area. Blumea. 1967; 15: 557–574.

[6] Kramer KU. The lindsaeoid ferns of the Old World V. The smaller Pacific islands. Blumea. 1970; 18: 157–194.

[7] Kramer KU. Flora Malesiana, series II. Pteridophyta. Vol. 1, part 3: *Lindsaea* Group. Groningen: Wolters-Noordhoff Publishing; 1971.

[8] Kramer KU. The lindsaeoid ferns of the Old World VI. Continental Asia, Japan and Taiwan. Gard Bull Singapore. 1972; 26: 1–48.

[9] Kramer KU. The lindsaeoid ferns of the Old World IX. Africa and its islands. Bull Jard Bot Natl Belg. 1972; 42: 305–343. 10.2307/3667462

[10] Kramer RU, Tindale MD. The lindsaeoid ferns of the Old World VII. Australia and New Zealand. Telopea. 1976; 1: 91–128. 10.7751/telopea19763202

[11] Tryon RM, Tryon AF. Ferns and Allied Plants with Special Reference to Tropical America. New York: Springer-Verlag; 1982.

[12] Ching RC. The Chinese fern families and genera, systematic arrangement and historical origin. Acta Phytotax Sin. 1978; 16 (3): 1–19, 16(4): 16–37.

[13] Smith AR, Pryer KM, Schuettpelz E, Korall P, Schneider H, Wolf PG. A classification for extant ferns. Taxon. 2006; 55: 705–431. 10.2307/25065646

[14] Hasebe M, Omori T, Nakazawa M, Sano T, Kato M, Iwatsuki K. *rbcL* gene sequences provide evidence for the evolutionary lineages of leptosporangiate ferns. Proc Natl Acad Sci USA. 1994; 91: 5730–5734. 10.1073/pnas.91.12.5730 8202555 PMC44070

[15] Schuettpelz E, Pryer KM. Fern phylogeny inferred from 400 leptosporangiate species and three plastid genes. Taxon. 2007; 56: 1037–1050. 10.2307/25065903

[16] Schuettpelz E, Pryer KM. Fern phylogeny In: Ranker TA, Haufler CH, editors. The biology and evolution of ferns and lycophytes. Cambridge: Cambridge University Press; 2008 pp. 395–416.

[17] Lehtonen S, Tuomisto H, Rouhan G, Christenhusz MJM. Phylogenetics and classification of the pantropical fern family Lindsaeaceae. Bot J Linn Soc. 2010; 163: 305–359. 10.1111/j.1095-8339.2010.01063.x

[18] Kramer KU. *Lindsaea* terrae-reginae, a new fern species from Queensland. Telopea. 1988; 3: 287–289. 10.7751/telopea19884817

[19] Holttum RE. A revised flora of Malaya II. Ferns of Malaya. Singapore: Government Printing Office; 1954.

[20] Brownlie G. The Genus *Lindsaea* in Fiji. Am Fern J. 1973; 63: 91–98. 10.2307/1546185

[21] Swofford DL. PAUP*: phylogenetic analysis using parsimony (and other methods). Sunderland, Mass: Sinauer; 2002.

[22] Murray MG, Thompson WF. Rapid isolation of high molecular weight plant DNA. Nucleic Acids Res. 1980; 8: 4321–4326. 10.1093/nar/8.19.4321 7433111 PMC324241

[23] Doyle JJ, Doyle JL. A rapid total DNA preparation procedure for fresh plant tissue. Focus. 1990; 12: 13–15.

[24] Taberlet P, Gielly L, Pautou G, Bouvet JP. Universal primers for amplification of three non-coding regions of chloroplast DNA. Plant Mol Biol. 1991; 17: 1105–1109. 10.1007/BF00037152 1932684

[25] Tate JA, Simson BB. Paraphyly of *Tarasa* (Malvaceae) and diverse origins of the polyploid species. Syst Bot. 2003; 28: 723–737.

[26] Sang T, Crawford DJ. Stuessy TF. Chloroplast DNA phylogeny, reticulate evolution, and biogeography of *Paeonia* (Paeoniaceae). Am J Bot. 1997; 84: 1120–1136. 10.2307/2446155 21708667

[27] Thompson JD, Higgins DG, Gibson TJ. CLUSTAL W: improving the sensitivity of progressive multiple sequence alignment through sequence weighting, position-specific gap penalties and weight matrix choice. Nucleic Acids Res. 1994; 22: 4673–4680. 10.1093/nar/22.22.4673 7984417 PMC308517

[28] Hall TA. BioEdit: a user-friendly biological sequence alignment editor and analysis program for Windows 95/98/NT. Nucleic Acids Symp Ser. 1999; 41:95–98.

[29] Simmons MP, Ochoterena H. Gaps as characters in sequence-based phylogenetic analyses. Syst Biol. 2000; 49(2): 369–381. 10.1093/sysbio/49.2.369 12118412

[30] Bozdogan H. Model selection and Akaike's information criterion (AIC): The general theory and its analytical extensions. Psychometrika. 1987; 52: 345–370. 10.1007/BF02294361

[31] Posada D. jModelTest: Phylogenetic model averaging. Mol Biol Evol. 2008; 25: 1253–1256. 10.1093/molbev/msn083 18397919

[32] Zwickl DJ. Genetic algorithm approaches for the phylogenetic analysis of large biological sequence datasets under the maximum likelihood criterion. Thesis, the University of Texas at Austin. 2006. Available: https://repositories.lib.utexas.edu/bitstream/handle/2152/2666/zwickld81846.pdf?sequence=2&isAllowed=y

[33] Ronquist F, Huelsenbeck J. MrBayes 3: Bayesian phylogenetic inference under mixed models. Bioinformatics. 2003; 19: 1572–1574. 10.1093/bioinformatics/btg180 12912839

